# Perspectives of Differentiation Therapies of Acute Myeloid Leukemia: The Search for the Molecular Basis of Patients’ Variable Responses to 1,25-Dihydroxyvitamin D and Vitamin D Analogs

**DOI:** 10.3389/fonc.2014.00125

**Published:** 2014-05-27

**Authors:** Aleksandra Marchwicka, Małgorzata Cebrat, Preetha Sampath, Łukasz Śnieżewski, Ewa Marcinkowska

**Affiliations:** ^1^Faculty of Biotechnology, University of Wroclaw, Wroclaw, Poland; ^2^Laboratory of Molecular and Cellular Immunology, Institute of Immunology and Experimental Therapy, Polish Academy of Science, Wroclaw, Poland

**Keywords:** cancer, differentiation, leukemia, all-*trans*-retinoic acid, 1,25-dihydroxyvitamin D_3_, vitamin D receptor, gene, exon

## Abstract

The concept of differentiation therapy of cancer is ~40 years old. Despite many encouraging results obtained in laboratories, both *in vitro* and *in vivo* studies, the only really successful clinical application of differentiation therapy was all-*trans*-retinoic acid (ATRA)-based therapy of acute promyelocytic leukemia (APL). ATRA, which induces granulocytic differentiation of APL leukemic blasts, has revolutionized the therapy of this disease by converting it from a fatal to a curable one. However, ATRA does not work for other acute myeloid leukemias (AMLs). Since 1,25-dihydroxyvitamin D_3_ (1,25D) is capable of inducing monocytic differentiation of leukemic cells, the idea of treating other AMLs with vitamin D analogs (VDAs) was widely accepted. Also, some types of solid cancers responded to *in vitro* applied VDAs, and hence it was postulated that VDAs can be used in many clinical applications. However, early clinical trials in which cancer patients were treated either with 1,25D or with VDAs, did not lead to conclusive results. In order to search for a molecular basis of such unpredictable responses of AML patients toward VDAs, we performed *ex vivo* experiments using patient’s blast cells. Experiments were also performed using 1,25D-responsive and 1,25D-non-responsive cell lines, to study their mechanisms of resistance toward 1,25D-induced differentiation. We found that one of the possible reasons might be due to a very low expression level of vitamin D receptor (VDR) mRNA in resistant cells, which can be increased by exposing the cells to ATRA. Our considerations concerning the molecular mechanism behind the low VDR expression and its regulation by ATRA are reported in this paper.

## Differentiation Therapy of Cancer

Cancer remains one of the leading causes of morbidity and mortality worldwide despite many improvements in diagnostic and therapeutic strategies. Development of cancer is a multi-step process which usually involves sustained proliferative potential, evading of growth-suppressing signals, resisting apoptosis, and enabling replicative immortality of the cell, due to gene aberrations and deregulated expression of genes (1). Recent development in the field of biological sciences along with a better understanding of the biology of cancer cell and its microenvironment, has led to new treatment methods. One such method is the targeted therapy, where new generation of cancer drugs is designed to interfere with a specific molecular target that is believed to have an important role in cell growth, survival, and differentiation and restore the differentiation capacity. This novel and less toxic form of therapy was designed to reprogram cancer cells, which resulted in the loss of proliferative capacity and induction of terminal differentiation or apoptosis of the cells (2). Classical chemotherapy involved killing of cells that divided rapidly (one of the main properties of cancer cells), which also killed cells that divided rapidly under normal circumstances, like bone marrow stem cells or cells in digestive tract. Hence, this method was highly toxic, which resulted in high death rates in cancer patients (3).

One particular type of cancer, with increasing incidence is acute myeloid leukemia (AML). AML is characterized by the accumulation of transformed primitive hematopoietic blast cells, which lose their ability of normal differentiation and proliferation (4). The studies conducted on characterization of the gene mutations and aberrant signaling pathways have provided insights into the mechanisms of leukemogenesis. Targeted therapy in AML was proposed in late twentieth century by Leo Sachs, which focused on forcing cancer cells to differentiate (5). This was a result of several *in vitro* analyses made in 1970s and 1980s, which showed that a number of compounds have anti-proliferative and pro-differentiating properties toward AML cells. For example, Breitman et al. showed that granulocytic differentiation of HL60 leukemic cells was possible after all-*trans*-retinoic acid (ATRA) treatment, which resulted in the rapid development of differentiation therapies and a better understanding of the differentiation mechanisms involved (6). This was because many cancer subtypes displayed alternations in the normal program of differentiation and growth. Hence, the use of specific agents that can trigger differentiation of leukemic cells along normal hematopoietic lineages became necessary (5).

## Retinoids in the Treatment of Acute Myeloid Leukemia

The above observations were translated into differentiation therapy, using ATRA in acute promyelocytic leukemia (APL) (7, 8). Pathogenesis of APL is associated with a chromosomal translocation that disrupts retinoic acid receptor α (RARα) gene located on the short arm of chromosome 17 (q21) and results in an arrest of the early stage of granulocytic differentiation (promyelocyte) (9). A balanced translocation between chromosomes 15 and 17 [t(15;17)(q22;q21)] (10), which leads to the formation of fusion of promyelocytic leukemia protein (PML) and RARα was found in 98% of APL patients. It was also shown that a small subset of patients carry other variants of 17q chromosome translocation (Table 1) (11, 12).

**Table 1:** **Variant translocations in APL [based on Ref. (11) and http://atlasgeneticsoncology.org/index.html]**.

Translocation	Translocation partner	Epidemiology
t(11;17) (q23;q21)	PLZF (promyelocytic leukemia zinc-finger protein)	1% of APL patients
t(5;17) (q35;q21)	NPM1 (nucleophosmin 1)	Exceptional (only two well-documented cases)
t(11;17) (q13;q21)	NUMA (nuclear mitotic apparatus)	Exceptional (only one case fully described)
der(17)	Stat5b (signal transducer and activator of transcription)	Exceptional (only one case fully described)

All cytogenetic aberrations found in APL are connected with RARα gene, which plays a central role in APL pathogenesis (11). Three forms of RAR, namely RARα, RARβ, and RARγ, are found to be important regulators of myeloid differentiation (13), which share structural similarity in their ligand and DNA-binding domains, but display distinct tissue-specific pattern of expression. When dimerized with a retinoid X receptor (RXR), RARs bind to specific promoter sequences upstream of genes (direct response elements – DRs) and promote their transcription in the presence of ATRA or 9-*cis*-RA. In the absence of a ligand, RAR/RXR heterodimers interact with nuclear receptor co-repressors (SMRT and N-CoR), that recruit histone deacetylases (HDACs) and induce chromatin condensation and repression of transcription (14).

Retinoic acid receptor α, a principal mediator of retinoic acid (RA) activity (15) regulates several genes involved in myeloid differentiation including transcription factor PU.1 (16, 17) and CCAAT/enhancer-binding proteins (C/EBPs): C/EBPβ (18) and C/EBPε (19). A second protein involved in the t(15;17) translocation is PML, a key organizer of nuclear bodies (NBs) that are bound to chromatin (20). Wild-type PML has a speckled nuclear pattern of expression, while PML/RARα has mostly a micropunctuated nuclear pattern or a cytoplasmic localization (21). PML regulates various cellular processes like defense against viral infection, DNA-damage, oxidative stress (22), regulation of transcription, heterochromatin remodeling, post-translational modification of proteins (23), and p53 signaling (24). PML is also an early mediator of transcription in myelopoiesis (25). Treatment of APL cells with RA caused reformation of normal NBs (21), which was caused by RA-induced degradation of a fusion protein, and in its absence the formation of NBs by proper PML protein resulting from the second, not mutated allele (26).

The fusion protein resulting from translocation t(15;17) always contains the N-terminal part of PML and the C-terminal part of RARα. The N-terminal part of PML is highly variable among patients and consists mostly of coiled-coil domain and dimerization domain. The C-terminal part of RARα contains dimerization, DNA, and ligand-binding domains and both transcriptional activating and repressing domains (27, 28). One of the most important features of the fusion protein as an oncogenic factor is the way it influences transcription. PML–RARα forms homodimers, through PML coiled-coil domains, which blocks the conformational changes associated with the release of co-repressors (SMRT and N-CoR), methyltransferase, and HDACs (29), thereby leading to histone H3 modifications (24). Thus, the altered RARα has lost its potential to respond to physiological concentrations of ATRA and acts as a constitutive repressor leading to RARα signaling block, resulting in inhibition of differentiation of APL cells (29). Moreover, PML–RARα is capable of antagonizing the transactivation function of wild-type RARα on RA-inducible promoters, and contributes to leukemogenesis by interfering with the process of differentiation (9).

Promyelocytic leukemia protein–RARα also heterodimerizes with wild-type PML, disrupting its localization in NBs (24). The best-known post-translational modification of PML is sumoylation. This modification stabilizes PML in NBs, which affects other PML-bound proteins and recruits transcriptional repressors (20). It is suggested that sumoylated PML in the fusion protein PML–RARα, might recruit repressors of transcription, leading to even stronger repression which cannot be reversed by physiological concentrations of ATRA (30). Experiments on mouse models have shown that the loss of second allele results in progression of the disease (31), which suggests that PML inactivation increases the transforming potential of PML–RARα by binding proteins involved in chromatin remodeling and transcriptional repression (32). In some cases of ATRA-resistant APL, the second allele of PML gene is mutated which causes accumulation of PML in cytoplasm, stabilization of PML–RARα, and inhibition of differentiation (33). The above data suggest that both RARα and PML play significant roles in the pathogenesis of leukemia (34).

In testing the role of PML–RARα in mediating the sensitivity of leukemic cells to ATRA, it was hypothesized that ATRA could influence C/EBP proteins, which are members of leucine zipper transcription factor family. Their ability to bind to DNA and regulate cell fate by mediating protein–protein interactions have been shown to be responsible for their effect on cell proliferation and differentiation (35). So far, C/EBPα, C/EBPβ, and C/EBPε have been reported to be involved at various stages of myeloid development and also responsible for transcription of genes important for blood cells’ functions (19, 36–42). Studies conducted on the mechanism of differentiation of APL cells by ATRA, demonstrated an increase in C/EBPβ RNA and protein levels (corresponding to increased C/EBPβ activity). This C/EBPβ activity was found to be controlled by PML–RARα, through C/EBPβ proximal promoter and was found to be absent in cells that were found to be resistant to ATRA (18). *In vitro* studies also demonstrated that transcription factor PU.1 was suppressed in cells carrying the PML–RARα fusion protein and that ATRA-induced granulopoiesis in these cells involved restoring the level of PU.1 (17). It appeared that induction of PU.1 by ATRA was possible only when the levels of C/EBP proteins were upregulated enough to activate the PU.1 promoter. This led to the conclusion that PU.1, an ATRA responsive gene is capable of overcoming the inhibition mediated by PML–RARα in the promoter region of PU.1 (17).

Recent experiments have shown that oncogenic transformation mediated by PML–RARα is a multi-step process, and does not take place just by repression of transcription. It was shown that unlike the wild-type RXR–RARα which recognizes DR1, DR2, and DR5 sequences, fusion protein PML–RARα has a broad range of response elements due to the presence of four DNA-binding sites (DR1–DR16) (43). Binding of RXR facilitates the binding of PML–RARα complex to widely spaced direct repeats resulting in a larger spectrum of controlled genes, causing a transcription deregulation in APL cells (43, 44). Moreover, it appeared that one of the *de novo* binding partners for the fusion protein is vitamin D receptor (VDR) and by sequestering it, PML–RARα causes the inhibition of 1,25-dihydroxyvitamin D_3_ (1,25D)-induced differentiation (45).

Treatment of APL patients with pharmacological doses of ATRA causes differentiation of APL cells toward granulocytes. At a molecular level, ATRA reverses the differentiation block by interacting with the ligand-binding domain of RARα, which leads to the release of co-repressors and the activation of genes responsible for transcription. After ATRA treatment, mature granulocyte-like cells enter programed cell death (46). Higher clinical efficiency in APL patients is achieved by a combination of ATRA with arsenic trioxide (ATO), which induces apoptosis through the mitochondrial pathway and by the formation of reactive oxygen species (ROS) (47). ATO has a limited differentiation capacity which is partial (48) and dose-dependent (49), most probably mediated by histone acetylation and transcriptional activation of many differentiation-related genes (50).

However, the most important effects of both ATRA and ATO treatment consist in degradation of PML–RARα (51). As it was presented in the past, wild-type RARα, as well as PML–RARα fusion, become degraded upon the treatment of the cells with RA (52). On the other hand, ATO targets the normal PML protein and PML–RARα fusion to proteasome-mediated degradation (53). This is mediated mostly by an ATO-induced oxidative stress, which leads to the cross-linking of PML proteins by disulfide bonds. PML consequently aggregates at the outer shell of NBs, the aggregates become massively sumoylated and are targeted to proteasome-mediated degradation (54). However, proteasome-mediated degradation is not the only mechanism of clearance of PML–RARα fusion protein in response to either RA or ATO treatment. As it was presented, both agents trigger the process of autophagy in APL cells, contributing to the degradation of the oncoprotein (55). Since both RA and ATO contribute to degradation of PML–RARα, their simultaneous use gives synergistic effects in clearance of APL blasts (51).

Also, clinical experiences have proven that ATRA and ATO treatments are synergistic and very efficient, converting APL from a fatal into a curable disease (56). When described for the first time, APL was considered the most aggressive form of AML, where death was caused predominantly by sudden hemorrhages resulting from coagulation disorders (57). The very first paper describing the use of ATRA to treat APL patients reported that all 24 patients involved in the study attained complete remission, which was revolutionary (7). Unfortunately, all of the patients from this initial group experienced a relapse of the disease (56) and, it was reported later that some of the patients, who relapsed after ATRA treatment, developed ATRA resistance (58). The next step forward led to the introduction of a combination therapy composed of ATRA and idarubicin, which led to molecular remission in 98% of patients, and 79% 2-year event-free survival rates (59). Although the remission rates obtained with ATRA and idarubicin were very high, the introduction of ATO (60) produced even better outcomes. The recent multicenter study, which compared the ATRA plus chemotherapy (idarubicin, mitoxantrone, methotrexate, and 6-mercaptopurine) with ATRA plus ATO in patients with APL, revealed that 2-year event-free survival rates were 97% in the ATRA–ATO group and 86% in the ATRA–chemotherapy group (61), showing that new paradigms of APL therapy should be introduced (62).

## Differentiation of Non-APL AMLs

Another type of AML where differentiation therapy might be useful is AML-M2 with translocation t(8;21)(q21;q22). This aberration leads to the fusion of acute myeloid leukemia 1 (AML1) protein and the eight-twenty-one (ETO) protein (63). Normal AML1 functions as a transcriptional activator, promoting granulocytic differentiation through the upregulation of lineage-specific target genes, while ETO protein (nuclear factor) possessing a self-associating domain, acts as a transcriptional repressor by recruiting HDAC complex (64). The fusion protein consists of the N-terminal DNA-binding domain of AML1 protein and almost entire ETO protein, with self-association and nuclear co-repressor interacting regions which influences multiple processes including myelomonocytic development (63). After fusion with ETO repressor protein, AML1 turns from being an activator to repressor which downregulates all of its target genes involved in granulocytic differentiation (64) through recruitment of co-repressors complex containing HDAC activity (65).

The AML1–ETO and PML–RARα share several common features, suggesting similar pathogenic mechanism in AML cells. Both are found to form oligomeric complexes with the increased affinity for HDACs, and act as transcriptional repressors of differentiation-related genes. RA signaling pathway is silenced in AML1–ETO AMLs through recruitment of HDACs on regulatory sites, resulting in the transcriptional silencing of the RARβ2 gene which leads to RA resistance and differentiation block (66). AML1–ETO also interacts with essential hematopoietic transcription factors such as C/EBPα, PU.1, and GATA1 and blocks their differentiation-promoting functions (64). The oncoprotein also interferes directly with RARα by binding to the receptor in a ligand-independent manner, thus blocking the ability of ATRA to mediate the co-regulator exchange and preventing activation of RARα target gene expression (63).

Novel differentiation agents for AML1–ETO positive AML are targeted toward HDACs, which are capable of modifying the histone acetylation status by removing acetyl groups, thus leading to chromatin silencing. Unlike chromosomal abnormalities or gene mutations which cause loss of gene function, silencing of the chromatin can be pharmacologically reversed by HDACs inhibitors. These compounds are promising agents that promote histone hyperacetylation, leading to chromatin relaxation and expression of genes important for normal cell growth and differentiation (67). HDACs inhibitors together with ATRA are used in ATRA-resistant APL with translocation PZLF–RARα (68), where HDACs sensitize cells to ATRA treatment (69). Since changing the silenced chromatin into an open form is a multi-step reaction, involving not only the release of HDACs but also the recruitment of chromatin remodeling complexes and transcriptional co-activators, the inhibition of HDACs seems to be only an initial step and is not sufficient enough to restore the complete cell differentiation (69). Therefore, a combination therapy could be beneficial for patients with AML1–ETO, where the RA pathway is repressed.

Another promising target in differentiation therapy is peroxisome proliferator activated receptor γ (PPARγ), which belongs to the nuclear receptor family and functions as a ligand-dependent transcription factor responsible for lipid metabolism (70). This nuclear receptor is a putative cancer therapy target, because it controls the expression of genes which are important for cell fate, for example genes involved in cell cycle regulation: p21Waf1/Cip1, p18INK4c (71), and β-catenin which is a key downstream component of the canonical Wnt signaling pathway (71, 72). The receptor is not only expressed in adipocytes but also in many other tissues and cell types throughout the body, including monocytes and macrophages (70). Furthermore, it was shown that the level of expression of PPARγ is higher in leukemic cell lines than in normal blood cells (73). Myelomonocytic leukemic cells express abundant PPARγ, and its ligands can force acute myelomonocytic leukemic cells to differentiate toward macrophages (74). It was shown that the PPARγ ligand (troglitazone) can inhibit clonal proliferation of myeloid monocytic leukemic cells U-937 when combined with ATRA and/or RXR ligands. All three tested compounds exhibited synergistic induction of differentiation with reduced viability of human myeloid leukemia cell lines (73).

Retinoid X receptor is yet another important target in AML. RXRs are receptors for vitamin A metabolites like 9-*cis*-RA and are the binding partners required for transcriptional activation by some other members of the steroid/thyroid hormone receptor superfamily, including RARs, VDR, and PPARs (75). RXR antagonists could become novel therapeutic agents for the treatment of AML. Studies using HL60 cells and leukemic patients’ cells showed that RXR antagonist (bexarotene) inhibits growth and induces differentiation of cells toward neutrophils. Phase I clinical trials using bexarotene in non-APL patients have shown that costimulation of both RAR and RXR receptors may be involved in differentiation of non-APL AML. It was suggested that differentiation in APL may occur through the RAR pathway while in non-APL similar effects may be achieved through RXR (76).

## 1,25-Dihydroxyvitamin D_3_

A very promising differentiation effect was demonstrated by the physiologically active form of vitamin D, namely 1,25D. The primary function of this secosteroid compound is to maintain the calcium and phosphorus homeostasis (77, 78). In addition, non-classical functions of 1,25D include: regulation of the immune response, influence on proliferation, maturation, and apoptosis of normal and neoplastic cells (77, 79–83). Several studies demonstrated the antitumor and pro-differentiating activity of 1,25D on cancer cells (82, 84–86), which led to early clinical trials to test the ability of 1,25D to treat AML and myelodysplastic syndromes (MDS) (82, 87).

The first discovery of the use of physiologically active form of vitamin D to induce the differentiation of AML promyeloid cell line HL60, into the cells resembling monocytes was done in 80s of twentieth century (88), during which it was shown that exposure to 1,25D can induce the ability of these cells to phagocytose and can inhibit proliferation. In due course, it was found that many other myeloid cell lines such as promonocytoid U937 and THP-1 also responded to 1,25D by exhibiting a decreased ability to proliferate and displaying many features of mature monocytes (89). MV4–11 and MOLM-13 cells, with mutated Fms-like tyrosine kinase 3 (FLT3) responded to 1,25D treatment by expressing cell differentiation markers, which was although lesser than in the model HL60 cell line (90).

On examining the mechanisms involved in the induction of differentiation by 1,25D, it was found that it exerts its effects by binding to a nuclear receptor called VDR. In the ligand-bound state, VDR heterodimerizes with RXR and attaches itself to the promoter regions of the target genes, such as osteocalcin (91), 24-hydroxylase of 1,25D (CYP24A1) (92, 93), kinase suppressor of Ras-1 (94), p27^Kip1^ (95), or CD14 (96) which play important roles in regulating the calcium–phosphate homeostasis, metabolism of 1,25D, cellular differentiation, and cell cycle. Except the well-known activation of VDR protein, which confers the so-called “genomic pathway of signal transduction,” 1,25D activates membrane initiated signaling pathways, known as “rapid responses,” such as MAPK pathways (97), the lipid signaling pathways (98), or PI3K–AKT pathway (99, 100). Induction of cell cycle arrest by influencing genes responsible for anti-proliferation, such as interferon α-inducible protein p27 (IFI27) (101) and p21 (102) has been found to be one of the main anti-proliferative actions of 1,25D. The pro-differentiating effect is mediated by transcription factors crucial for myeloid hematopoiesis and includes transient upregulation of C/EBPα and sustained upregulation of C/EBPβ (103). The initiation of differentiation in HL60 cells, was observed by a rapid growth followed by growth arrest and accompanied by a monocytic differentiation (104). This was in common with U937 cells which after initial proliferative burst, later showed elevated levels of cyclin-dependent kinase inhibitors, p21^Waf1/Cip1^ and p27^Kip1^ (105). Other mechanisms include a decrease in the level of gene expression of exportin and importins (which are involved in regulating the traffic of substrates for protein production) (106) and elf2 (a translation initiation factor) (107) in HL60 cells suggesting decreased protein synthesis and suppression of translation.

## Clinical Trials Using Vitamin D Compounds in the Treatment of Acute Myeloid Leukemia

The *in vitro* studies described above led to nine studies, which examined the effects of different vitamin D compounds, such as 1,25D or 1-hydroxyvitamin D_3_ (alfacalcidol; precursor of 1,25D) in AML patients, either alone or in combination with other agents such as cytarabine, RA, hydroxyurea, or interferon α. These studies were conducted on small groups ranging from 1 to 29 patients with AML, where vitamin D compounds induced partial differentiation and the results were modest (Table 2) (108–116). It is important to note that all these studies used different dose schedules and some patients developed hypercalcemia, which was recently exhaustively reviewed (117). The doses of vitamin D compounds ranged from 0.5 to 15 μg/day in the above clinical trials. In the first clinical trial, alfacalcidol was used at the doses ranging from 4.5 to 15 μg/day and two out of three patients (one patient suffered from MDS) developed transient hypercalcemia which showed that this is a common side effect of vitamin D treatment (109). In the later studies, alfacalcidol and 1,25D were administrated at doses from 0.25 to 1 μg/day to avoid hypercalcemia (117). This reinforced the need to synthesize vitamin D analogs (VDAs) with decreased calcemic activity and greater antitumor activity (85, 118, 119).

**Table 2:** **Clinical trials using vitamin D compounds in AML patients (117)**.

Vitamin D compound	No. of patients	Response	Reference
Alfacalcidol	2	Transient hypercalcemia, decrease of blasts in bone marrow	Irino and Taoka (109)
Alfacalcidol	2	One minor response in AML M4 patient	Takahashi et al. (111)
Alfacalcidol	1	One major response	Nakayama et al. (110)
Alfacalcidol, cytarabine, INFα, retinoids	15	5	Hellström et al. (108)
Alfacalcidol, cytarabine, 13-*cis*-retinoic acid	15	5	Hellström et al. (112)
1,25D, cytarabine, 13-*cis*-retinoic acid	26	15 Response: 58%	Ferrero et al. (116)
1,25D, cytarabine, hydroxyurea	29	13 Complete remissions, 10 partial remissions, response: 79%	Slapak et al. (115)
Alfacalcidol	11	17% Complete remission, 45% partial remission, response: 18%	Petrini et al. (113)
Alfacalcidol	21	Complete remission 17%	Petrini et al. (114)

Several *in vitro* studies have shown that the biological effects of 1,25D can be selectively modulated in combination with other drugs (86, 120, 121). Slapak et al. presented promising results from the study conducted on 29 patients who were treated with cytarabine, hydroxyurea, and 1,25D, showing an overall response of 79% where 45% was complete remission and 34% was partial remission. Hence, it was proposed that favorable results could have been due to the synergistic effect of the tested compounds (115). It has been shown that 1,25D activity can be potentiated in prostate cancer by using CYP24A1 inhibitors (122, 123). CYP24A1 is involved in the catabolism of 1,25D (124) and its overexpression is associated with poor prognosis of some human cancers (125–127).

These early clinical trials using high doses of 1,25D raised an important issue of the maximum tolerable dose in clinical use. It was proposed that a maximum dose of 1,25D is >100 μg/week intravenously and an oral dose of 0.15 μg/kg/week. Commercially available 1,25D can be used in high doses for oral administration, however intravenous administration with high levels gives the best therapeutic results (102). Several studies revealed that pharmacokinetic dose escalation did not result in the escalation of systemic exposure. Desirable linear relationship between dose and systemic exposure was lost at doses >16 μg/kg/week (128–130).

## Variable *Ex Vivo* Responses of AML Patient’s Blasts toward 1,25D or VDA-Induced Differentiation

There might be multiple reasons for the unsatisfactory results of the above mentioned clinical treatments of AML patients using 1,25D or its analogs. One very important reason is that the patients with AML were not stratified further into subgroups, despite the fact that AML is not just one disease, but a heterogeneous group of diseases with various underlying causes. Traditional classification of AML introduced by French–American–British (FAB) hemato-oncologists, which was based on morphology, immunochemistry, and immunophenotyping of blast cells, divided AML into eight subgroups (131). More recently, a new classification was proposed, which includes cytogenetics and molecular diagnostics (132). So far, there are more than 200 distinct mutations reported in AML blasts (133), however some of them are more frequent than the others.

Some of the mutations found in AML blasts have their independent prognostic value, and therefore routine molecular genetics analyses are implemented in diagnostic procedures nowadays (134). The mutation, which has the highest prognostic impact is the above mentioned t(15;17), which leads to the fusion protein PML–RARα. This mutation is characteristic of M3 subtype of AML (according to FAB classification), the subtype which renders this leukemia susceptible to ATRA treatment and confers almost 80% survival after 10 years from diagnosis (135). Another example of an abnormality with a good prognostic value is mutations in nucleophosmin 1 (NPM1), which are present in about 30% of AML patients. NPM1 is a protein which normally shuttles between cytosol and nucleus where it chaperons transport of other proteins, but when mutated is retained in the cytosol of the cell (136, 137). For patients with NPM1 mutations, the overall survival rate after 10 years is about 52% (135). The good prognosis for the above patients is limited to the individuals with wild-type FLT3. In contrast, FLT3 mutations and specifically internal tandem duplications (ITD), confer a bad prognosis for patients (135). Wild-type FLT3 is important in normal hematopoiesis, where its activation is strictly regulated by the FLT3 ligand. FLT3–ITD mutation causes constitutive activation of the receptor and enhanced proliferative potential of blast cells (138). This mutation is present in 20–27% of AML patients, whose overall survival rate after 10 years is only 10% (135). The above examples show clearly how high is the impact of individual mutation for the blast cell fate. The overview of the prognostic significance of selected recurring chromosomal and molecular abnormalities based on the study conducted on younger adult patients (135, 139) is given in Table 3. It should be remembered that the combinations of multiple mutations are very common in AML patients (134) and that the higher number of abnormalities carried by a patient worsens an individual prognosis (139).

**Table 3:** **Impact of selected cytogenetic abnormalities on disease outcome based on Ref. (135, 139)**.

Cytogenetic or molecular abnormality	Fusion protein or mutated protein	OS after 10 years (%)
t(15;17)	PML–RARα	77–81
t(8;21)	AML1–ETO	61–65
CEBPA biallelic	C/EBPα	51
FLT3 wt; NPM1 mut	Nucleophosmin	52
t(9;11)	MLLT3–MLL	39–59
t(6;9)	DEK/NUP214	27–29
FLT3–ITD; NPM1 wt	FLT3–ITD	10
−7/del(7q)	–	10
−5/del(5q)	–	6

*OS, overall survival*.

Therefore, we hypothesized that the mutations present in AML blasts could also influence their susceptibility to 1,25D-induced monocytic differentiation. In order to test this hypothesis, we performed two sets of experiments using blasts from the peripheral blood of freshly diagnosed AML patients. These blasts were *ex vivo* exposed to either 1,25D or low calcemic VDAs and then tested using flow cytometry for the presence of monocytic differentiation marker CD14. In the first series of experiments, the blast cells from 32 patients (140), while in the second series from 56 patients (141) were studied. The analysis of data revealed that the cells with mutated FLT3 were less responsive to 1,25D-induced differentiation than the remaining blast cells (140, 141). Therefore, we wanted to further investigate the nature of that correlation. For this purpose, we used two AML cell lines, which carry FLT3–ITD mutations: MV4–11 cell line has mutation in both, while MOLM-13 in one allele (142). Our *in vitro* experiments revealed that these two cell lines were responsive to 1,25D and to VDAs, which pointed out that the correlation observed in AML patients blasts with FLT3 mutation, the low differentiation response was not caused by mutation itself, but by other, unknown to us at this moment reasons (90). In our *ex vivo* experiments, we also observed that the blast cells from AML patients, having NPM1 mutation had a tendency to differentiate better than other blasts. However, many patients have both of the above mutations and in our experiments, the blasts with both mutations differentiated better in response to 1,25D than the blasts with FLT3–ITD, but weaker than the ones with mutated NPM1 alone.

In our *ex vivo* experiments, we also observed that deletion of a short arm or whole of chromosome 7 [−7/del(7q)] in AML blast cells correlated with a stronger differentiation response, in comparison with other blast cells. Thus, we hypothesized that there could be a gene located in the short arm of chromosome 7, which is responsible for degradation of 1,25D. The gene which supposedly could take part in such process is the one which encodes NADPH-cytochrome P450 reductase (CYPOR), an enzyme responsible for electron transfer to cytochrome P450, and vital in the metabolism of drugs and steroid production in humans. From our further experiments, we documented that indeed the expression of this gene and the level of an enzyme in AML cells was strongly upregulated by RA, but also to some extent by 1,25D (143). Another conclusion which could be drawn from our experiments is that blasts from only few patients differentiate in response to 1,25D as strongly as model AML cell lines, such as HL60, MV4–11, MOLM-13, U-937, or NOMO-1 (90, 140, 144). Together, our results indicate that responses of individual patients’ blast cells to 1,25D are variable and that due to the heterogeneity of the disease and overlapping mutations, statistical analysis is very difficult. In order to find the impact of individual mutation, very big groups of patients should be included in the *ex vivo* tests. Moreover, one should remember that *ex vivo* tests are fundamentally simple in comparison to the clinical trials, where the AML blast cells, which should get differentiated in response to 1,25D or its analogs, are present in a living body with an immune system and other accompanying diseases. This explains why the small clinical trials described above produced so many inconclusive results.

## RA and 1,25D Combination Treatments

The idea of using 1,25D or VDAs in combination with retinoids to elicit better anti-tumor effects, than treating patients with 1,25D or RA alone to produce synergistic effects was suggested in the past (86). The studies were conducted on myeloblastic cell line (HL60) and APL cell line (NB4) using VDA: 20-epi-22oxa-24a,26a,27a-tri-homo-1,25(OH)_2_D_3_ (KH1060) and 9-*cis*-RA, which promoted the differentiation and inhibited the growth of the cell lines. This was found to be achieved by reduced anti-apoptotic bcl-2 and increased pro-apoptotic bax expression (145, 146). Few other VDAs like 1,25-(OH)_2_-δ-16-23-yne-cholecalciferol and 1,25-(OH)_2_-23-yne-cholecalciferol, were found to promote much more differentiation than 1,25D, with very little effect on the calcium-phosphate homeostasis. When used in combination with RA *in vitro* on HL60 cells, an additive pro-differentiating effect was observed (147). When 9-*cis*-RA was used with 1,25D in combination therapy, which induced granulocytic and/or monocytic differentiation, TGF-β1 was identified as the secondary mediator in AML cells (148).

## The Search for a Molecular Basis of an RA-Regulated Transcription of VDR

One of the AML cell lines being used in our laboratory is KG-1, which is unresponsive to 1,25D or VDAs (149, 150), but responsive to RA. As presented before, RA inhibited clonal growth in KG-1 to a higher degree than in HL60 cells (6, 151) and the inhibitory effect of RA on the growth of KG-1 cells was irreversible even after its removal from the cells (151). We documented from our experiments that these cells respond to ATRA with upregulation of VDR gene expression, which restores their sensitivity to 1,25D. Constitutive expression of VDR gene was 10–12 times lower in KG-1 cells, in comparison with 1,25D-responsive HL60 cells, and after ATRA treatment it increased by about 8 times. Surprisingly, our experiments documented that HL60 cells respond to ATRA with downregulation of VDR gene expression (150).

The gene which encodes human VDR was cloned in 1988 (152). This gene is located on chromosome 12 and spans about 100 kb of genomic DNA. Translation of VDR protein starts from exon 2, and due to T to C polymorphism which eliminates the most 5′-located ATG codon, the translation starts from the second in-frame ATG codon in some individuals. As a result, two variants of VDR protein exist, one three amino-acids shorter (424 aa) than the other (427 aa) (153), and the shorter variant was found to exert higher transcriptional activity due to a better contact with the human basal transcription factor IIB (TFIIB) (154, 155). Knowing that, we hypothesized that low activity of 1,25D in KG-1 cells, and high activity in HL60 cells, might have resulted from the different VDR protein variants in these cells. Therefore, the exon 2 was sequenced in the above cell lines, and the results indicated that KG-1 cell line is indeed homozygous in two longer variants of VDR, while HL60 cells have one longer and one shorter variant. However, U-937 cell line which was also found to be homozygous in two longer variants of VDR, differentiated *in vitro* in response to 1,25D or VDAs, showing that length of VDR is not the only reason for KG-1 cells’ resistance to 1,25D. The remaining cell lines used in our laboratory, namely NB4, MV4–11, MOLM-13, and NOMO-1 were found to be heterozygous with respect to VDR protein length, while THP-1 is homozygous in two shorter variants of VDR (our unpublished data).

**Figure 1:**
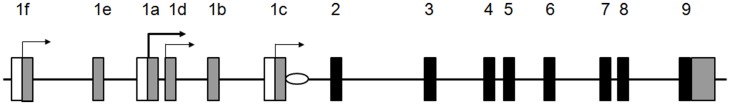
**Organization of human VDR locus**. Black boxes represent protein coding exons, gray – non-coding exons localized in the regulatory region of the gene. Horizontal arrows indicate transcriptional start sites. White boxes represent promoter regions, white oval – the RA-responsive *cis*-regulatory element.

Characterization of factors that regulate expression of the VDR gene at the transcriptional level is hampered because of the complexity of the promoter region. To date, several small non-coding exons have been identified (exons 1a–f) in the large regulatory region encompassing 65 kb upstream of the so-called coding region of VDR gene (exons 2–9) (Figure 1). Numerous combinations of the 5′-UTR of VDR are formed by an alternative splicing and/or different promoter usage. The main promoter of the VDR gene is the region associated with exon 1a (153). This promoter is characterized by the lack of canonical TATA- and CAAT-box and the presence of multiple specificity protein 1 (Sp1)-binding sites in a GC-rich region localized ~100 bp upstream of the transcriptional start site (TSS) present in exon 1a. This promoter region contributes to the constitutive VDR expression in multiple tissues and cells and has been shown to be predominant in human kidney, intestine, and bone (156). The 1a promoter region not only regulates the transcripts originating from exon 1a but also from exon 1d (156) which can be a result of the imprecise TSS localization typical for the TATA-less promoters. Exon 1d contains an alternative ATG codon and transcripts containing this exon have the potential to encode N-terminally extended VDR isoforms (VDRB1 and VDRB2) which have been shown to be co-expressed with the canonical VDR isoform (VDRA) (157, 158).

In contrast to the constitutive activity of 1a promoter region, the promoter associated with exon 1f, localized in the furthest distance from the coding region, drives expression of transcripts which have been shown to be tissue and cell-type specific. Transcripts originating from exon 1f are present in kidney tissue, parathyroid adenoma tissue, and in intestinal cell line (LIM 1863), which, as it has been noticed in the original work, represent the major target for calcitropic effects of 1,25D (156). The 1f promoter has been reported to contain sequences binding multiple transcriptional factors such as cAMP response element-binding protein (CREB), Wilms tumor protein (Wt-1), and Caudal type homeobox 2 (Cdx-2) (159, 160). In contrast to cell- and tissue-restricted expression of the corresponding transcripts, the 1f promoter region, when fused to reporter genes alone, displays its activity in variety of cell types which might suggest the existence of a distal regulatory region associated with the 1f promoter and regulating its tissue-specific activity.

In view of the scope of this review, the most interesting issue is the responsiveness of the VDR promoter(s) to hormonal regulation, especially by RA. Unlike the above described promoter regions (1f and 1a), the TATA-containing promoter associated with 1c exon (proximal to the coding region of VDR) has proven to be regulated by 1,25D, RA, estrogens, and phytoestrogens which upregulate the promoter activity in breast cancer cell lines (161). The sequence of 1c promoter does not contain any estrogen or 1,25D response elements but, similar to promoter 1a, contains Sp1 binding sites localized in GC-rich region. Three Sp1 consensus binding sites were identified to independently confer responsiveness to 1,25D, estrogens, and resveratrol (162).

According to our observations, the regulation of the activity of 1c promoter cannot be the only explanation for the different transcriptional activity of VDR gene observed in HL60 and KG-1 in response to RA, because the predominant types of VDR transcripts observed in these cells contain exon 1a which indicates the use of the 1a promoter region or promoter(s) further upstream exon 1a in regulating VDR expression in these cells. This would suggest that the regulatory element that confers the responsiveness to RA may not be localized in the promoter itself but rather in a *cis*-element cooperating with the promoter. One such element had been discovered during the initial characterization of the VDR promoters and is localized in an intron between exon 1c and exon 2 of VDR gene (153). This element has been shown to mediate increased activity of 1c promoter in response to RA and, more interestingly, was able to transfer the RA-response to an unrelated viral promoter proving its potential to regulate other promoters in the locus. It will be very interesting to fully characterize this element and perhaps to identify other enhancers/silencers present in VDR regulatory region and their interactions with the VDR promoters. This area of research seems to be somewhat neglected, probably due to the complexity and the size of the VDR regulatory region but the aid of techniques enabling the characterization of interactions between distal DNA domains, such as chromosome conformation capture (163) should provide new clues to fully characterize the regulation of VDR transcription in different cell types. Since we suppose that finding the mechanism of transcriptional up- and down-regulation of VDR gene in response to ATRA might be relevant for differentiation therapy of AML patients, the search for the regulatory elements in VDR gene is underway in our laboratories.

## Conflict of Interest Statement

The authors declare that the research was conducted in the absence of any commercial or financial relationships that could be construed as a potential conflict of interest.
